# Paternal reprogramming-escape histone H3K4me3 marks located within promoters of RNA splicing genes

**DOI:** 10.1093/bioinformatics/btaa920

**Published:** 2020-11-23

**Authors:** Nan Hao, Huawei Xin, Xiaowei Shi, Jie Xin, Haijuan Zhang, Shaofen Guo, Zhen Wang, Chunxiang Hao

**Affiliations:** College of Pharmacy, Linyi University, Linyi 276000, China; School of Mathematics and Statistics, Wuhan University, Wuhan 430072, China; College of Pharmacy, Linyi University, Linyi 276000, China; College of Pharmacy, Linyi University, Linyi 276000, China; College of Pharmacy, Linyi University, Linyi 276000, China; College of Pharmacy, Linyi University, Linyi 276000, China; College of Pharmacy, Linyi University, Linyi 276000, China; College of Pharmacy, Linyi University, Linyi 276000, China; College of Pharmacy, Linyi University, Linyi 276000, China

## Abstract

**Motivation:**

Exposure of mouse embryos to atrazine decreased histone tri-methylation at lysine 4 (H3K4me3) and increased expression of alternatively spliced RNA in the third generation. Specificity protein (SP) family motifs were enriched in the promoters of genes encoding differentially expressed alternative transcripts.

**Results:**

H3K4me3 chromatin immunoprecipitation sequencing (ChIP-seq) of mouse sperm, preimplantation embryo development and male gonad primordial germ cells (PGCs) were analysed to identify the paternal reprogramming-escape H3K4me3 regions (RERs). In total, 251 RERs selected harbour H3K4me3 marks in sperm, with signals occurring in the paternal genome during early development and in male gonad PGCs, and 179 genes had RERs within 1 kb of transcription start sites (TSSs). These genes were significantly enriched in the gene ontology term ‘RNA splicing’, and SP1/SP2/SP3 motifs were enriched in RER-associated H3K4me3 peaks. Overall, the H3K4me3 marks within TSSs of RNA splicing genes survived two rounds of the epigenetic reprogramming process.

**Supplementary information:**

Supplementary data are available at *Bioinformatics* online.

## 1 Introduction

Histone modifications have been demonstrated to be tightly associated with environmental exposures, and these modifications can be transmitted from sperm to the next generation, thereby affecting the offspring’s phenotype (Puschendorf *et al.*, 2008; Siklenka *et al.*, 2015). Exposure to the herbicide atrazine (ATZ) or the pesticide chlordecone leads to transgenerational effects, and these changes are related to changes in histone trimethylation at lysine 4 (H3K4me3) (Gely-Pernot *et al.*, 2018; Hao *et al.*, 2016).

Despite growing evidence supporting the heritability of histone modifications, the process of transmitting histone changes from mammalian gametes to the subsequent generation is poorly understood (Skvortsova *et al.*, 2018). Specifically, the heritability of epigenetic modifications is highly restricted by two rounds of epigenetic reprogramming events during the mammalian life span (Lange and Schneider, 2010; Zheng *et al.*, 2016).

The first phase of genome-wide epigenetic reprogramming occurs during early embryonic development, which is initiated immediately after fertilization (Zhang *et al.*, 2016). Although the majority of the H3K4me3 peaks are depleted in zygotes, some of the paternal H3K4me3 peaks within promoters of zygotes indicate basal enrichment and resemble the pattern of the late two-cell embryos. These findings suggest that these domains may include any H3K4me3-modified nucleosomes transmitted from sperm.

Mouse primordial germ cells (PGCs) migrate to the genital ridge by embryonic day 11.5 (E11.5), whereupon the second phase of genome-wide epigenetic reprogramming takes place. It was reported that a small number of specific genomic areas resist demethylation and thus retain their epigenetic markers (Seisenberger *et al.*, 2013; Xavier *et al.*, 2019). The reprogramming events that occur in early embryonic development and in PGCs constitute the most intense period of epigenetic change during the mammalian life cycle (Skvortsova *et al.*, 2018). Any epigenetic modifications that escape both of these reprogramming events can persist throughout the life of the individual and may be passed down to future generations (Ng *et al.*, 2010). Thus, errors in the response to external environmental factors that fail to be corrected by normal epigenetic maintenance processes or reprogramming events may lead to epigenetic abnormalities in the offspring.

The results of our previous study showed that embryonic exposure to ATZ led to a global decrease in H3K4me3 marks and an increase in the expression of alternatively spliced RNA isoforms. In addition, the 500 bp upstream sequences corresponding to the promoters of genes active in testis tissue encode differentially expressed alternative transcripts in the F3 generation, and the different peaks in the F3 generation were enriched in SP family motifs (Hao *et al.*, 2016). To further inspect the underlying mechanism involved in the inheritance of H3K4me3 marks between generations, in this study, we examined H3K4me3 marks that endure both rounds of epigenetic reprogramming events during embryonic development in sperm. The role of these domains in epigenetic inheritance was investigated. To identify the paternal reprogramming-escape H3K4me3 regions (RERs), large-scale H3K4me3 chromatin immunoprecipitation sequencing (ChIP-seq) datasets of all seven stages of preimplantation embryonic development and male gonad PGCs (E11.5 and E13.5) were analysed. A total of 251 RERs that harbour H3K4me3 marks in sperm were selected, and these signals persist in the paternal genome during early development and in male gonads of PGCs. A total of 179 genes were identified as having RERs located within 1 kb of their transcription start site (TSS). Importantly, the RER-related genes were significantly enriched in Gene Ontology (GO) terms such as ‘RNA splicing’, ‘mRNA processing’ and ‘gene expression’. In addition, H3K4me3 peaks, including RERs, were enriched in SP1/SP2/SP3 and SRY motifs. These results suggest that H3K4me3 marks within the TSSs of splicing genes may occur for genes that are sensitive to embryonic exposure to ATZ. The altered marks retained during two rounds of epigenetic reprogramming are transferred to subsequent generations and contribute to affecting the appearance of differentially expressed new isoforms in the third generation. The transcription factors SP1/SP2/SP3 and SRY may be involved in regulating the expression of these new isoforms.

## 2 Materials and methods

### 2.1 Datasets

The genome-wide histone modification H3K4me3 chromatin immunoprecipitation sequencing (ChIP-Seq) data of sperm and the paternal genome of mice during early development [including pronuclear stage 5 (PN5) zygotes, the two-cell stage, the four-cell stage, eight-cell embryos and inner cell mass (ICM)] were downloaded from the NCBI Gene Expression Omnibus (GEO) (Accession No. GSE71434) (Zhang *et al.*, 2016). ChIP-Seq data for H3K4me3 for the mouse embryonic day 11.5 (E11.5) and 13.5 (E13.5) male gonad PGCs were obtained from GEO (Accession No. GSE38164) (Ng *et al.*, 2013). Comprehensive RNA-seq data for primitive spermatogonia (PriSG), type-A spermatogonia (SG-A), type-B spermatogonia (SG-B), leptotene spermatocytes (lepSC), pachytene spermatocytes (pacSC), round spermatids (rST) and elongating spermatids (eST) were retrieved from the Gene Expression Omnibus database (series accession number GSE35005) (Gan *et al.*, 2013).

The mouse testis, liver and brain RNA-seq data from the F3 generation were obtained from progeny of gestating mice that were treated with ATZ during the embryonic period (E6.5 to E15.5) (Accession No. GSE81093) (Hao *et al.*, 2016).

### 2.2 Identification of RERs

Information concerning the sperm H3K4me3 peaks and paternal H3K4me3 peaks at 6 early developmental stages [pronuclear stage 5 (PN5) zygote, early 2-cell stage, late 2-cell stage, 4-cell stage, 8-cell embryo and inner cell mass (ICM)] were downloaded from the Gene Expression Omnibus (GEO) database (Accession No. GSE71434). The H3K4me3 peaks from male gonad PGCs (E11.5 and E13.5) were identified based on the following steps. First, the sequences from fastq files were mapped back to the mouse mm9/Ensembl genome using Bowtie 2.3.4, with a seed length of 22. Only tags that passed the quality filter and that mapped uniquely to the genome were used. Second, H3K4me3 peaks were identified using MACS 2.1.1 (Feng *et al.*, 2012), with the corresponding input and a false discovery rate (FDR) threshold less than 0.05. The BEDtools program with function intersection was used to determine the RERs (Quinlan and Hall, 2010), which were defined as regions with H3K4me3 signals appearing at all nine stages.

### 2.3 Classification of RERs as ‘enhancers’ or ‘promoters’

To classify the RERs as enhancers or promoters, we used a published dataset that comprised both H3K4me3 and H3K4me1 ChIP-seq data of mouse embryonic stem cells (Yu *et al.*, 2013). For each RER, we calculated the intensities of H3K4me3 and H3K4me1 marks from mouse embryonic stem cells. The RERs that showed higher intensities of H3K4me3 than H3K4me1 were defined as ‘promoters’, and the RERs with H3K4me1 intensities higher than those of H3K4me3 were considered ‘enhancers’.

### 2.4 Identification of RER-associated genes and gene functional annotation

Genes with RERs located within 1 kb of the TSS were selected with GREAT (version 3.0.089 and version 4.0.4), with the default parameters (McLean *et al.*, 2010). GO term analyses were performed using PANTHER, with an FDR < 0.05 (Ashburner *et al.*, 2000; Mi *et al.*, 2019; The Gene Ontology, 2019).

### 2.5 Motif enrichment analysis

The repeat masked sequences of RERs involving H3K4me3 peaks were used for analysis. Motif identification was performed using MEME-ChIP (Machanick and Bailey, 2011) with the default parameters. The identified motifs were compared with known motifs using TomTom (Gupta *et al.*, 2007) with the default parameters, and Find Individual Motif Occurrences (FIMO) was used to scan for SP1/2/3 and SRY motif-binding sites (Grant *et al.*, 2011). Mammalian conservation scores for each motif-binding site were obtained from the PhastCons30wayPlacental table from the UCSC Genome Browser website. Highly conserved binding sites (>0.70 conservation score) were retained as potential targets.

### 2.6 Identification of differentially expressed genes

The transcript expression profiles were generated from the RNA-seq data as described previously (Hao *et al.*, 2016). To identify whether any RER-associated genes were differentially expressed between the ATZ- and control-derived samples, we first selected the transcripts derived from the RER-associated genes, after which we filtered the transcripts with a more than 2-fold difference between the ATZ- and control-derived samples. Last, we employed a statistical test implemented in the R package Limma, with an FDR of less than 0.1 (Ritchie *et al.*, 2015).

## 3 Results

### 3.1 Identification of paternal epigenetic RERs

In mammalian embryos, there are two cycles of epigenetic reprogramming of the genome: the first phase is initiated immediately after fertilization, and the second phase takes place during PGC development. Extensive epigenetic reprogramming occurred for paternal H3K4me3 after fertilization (Zhang *et al.*, 2016); thus, we sought to determine if there are any H3K4me3-modified nucleosomes transmitted from sperm to early embryos. Analysis of the mouse sperm and genome-wide H3K4me3 ChIP-seq data of the paternal genome of mice during early development [including pronuclear stage 5 (PN5) zygotes, the two-cell stage, the four-cell stage, eight-cell embryos and ICMs from blastocysts] revealed 203 genomic regions with retained H3K4me3 marks in the paternal genome from sperm to mouse early development stages (Supplementary Table S1). These data suggest that the H3K4me3 marks within these 203 genomic regions may escape paternal genome epigenetic reprogramming after fertilization.

**Fig. 1.**
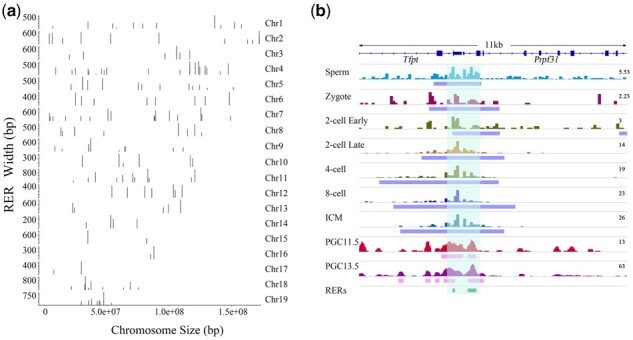
Identification of RERs. (**a**) The genome-wide distribution of RERs: the *X*-axis shows the chromosome coordinates; the *Y*-axis shows the width of the RERs. The chromosome numbers on the right and the black bars show the positions of the RERs on the chromosomes. (**b**) View of H3K4me3 enrichment in sperm, the paternal allele of early embryos and PGCs (E11.5 and E13.5). The H3K4me3 marks were visualized via IGV genome viewer version 2.7.2

The paternal reprogramming-escape H3K4me3 regions (RERs) were defined as regions with H3K4me3 marks in sperm and at all six stages of early embryo development in the paternal genome (including PN5 zygotes, the early two-cell stage, the late two-cell stage, the four-cell stage, eight-cell embryos and ICM) and 2 stages of PGC development in the male gonads (including E11.5 and E13.5). To further detect the genomic regions that have H3K4me3 marks in the paternal genome from sperm to mouse early developmental stages and that can escape the second round of epigenetic reprogramming during PGC development, H3K4me3 peaks from E11.5 and E13.5 male gonad PGCs were identified. Approximately 40 thousand and 100 thousand peaks (*q*-value cut-off = 0.05, generated by MACS 2.1.1) were called from the E11.5 and E13.5 PGCs, respectively. Remarkably, the H3K4me3 peaks from the PGCs were much narrower (with a median width of approximately 250 bp) than the peaks from the early embryonic developmental stages (with a median width of approximately 2000 bp, Supplementary Fig. S1a). The H3K4me3 peaks from the E11.5 and E13.5 PGCs were then compared with the 203 genomic regions that escaped the early development epigenetic reprogramming in the paternal genome. Interestingly, 251 genomic regions were identified with H3K4me3 marks that were not only retained in sperm and early embryonic developmental stages but also persistent during both E11.5 and E13.5 PGCs (Supplementary Table S2). The number of regions increased since the median width of the 203 regions was 1029 bp, while the median width of the 251 regions was 252 bp (Supplementary Fig. S1b). The locations of the region throughout the whole genome are shown inFigure 1a. For example, the *Brca2* gene, which is involved in double-strand break repair and homologous recombination and the *Prpf31* gene retained H3K4me3 marks within the TSS in sperm, and the peaks survived two rounds of epigenetic reprogramming (Fig. 1b).

### 3.2 The RERs are located close to gene TSSs

**Fig. 2.**
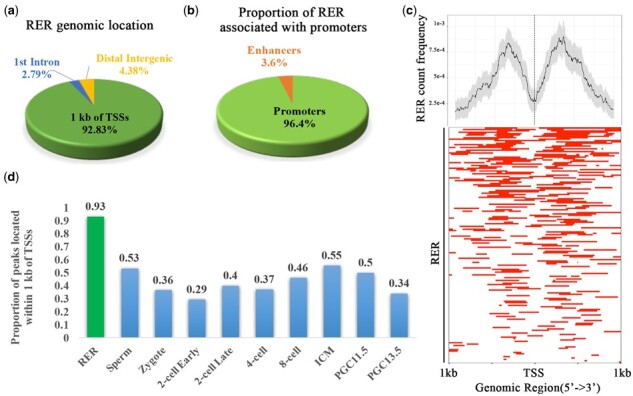
Genomic distribution of RERs. (**a**) Pie chart showing the distribution of RERs across gene features (within a ±1 kb window around TSSs, introns and intergenic regions). (**b**) Pie chart showing the classification of RERs as promoters or enhancers. (**c**) Top, Average profile of RERs found within ±1 kb around TSSs [the confidence interval was estimated by the bootstrap method (resample = 1000)]. Bottom, Heatmap of RERs found within ±1 kb around annotated gene TSSs. (**d**) Proportion histogram showing peaks located within ±1 kb around TSSs, with each bar representing one stage (*P*-value < 2.2e-14, Chi-square test)

Next, we investigated the localization of RERs with respect to gene regulatory features such as TSSs, exons, introns and intergenic regions. Interestingly, the majority (92.83%) of RERs were found within 1 kb of any TSS, while a small fraction of the RERs were either intronic (2.79%) or intergenic (3.98%) (Fig. 2a). The ratio of H3K4me3 to H3K4me1 indicates the tendency of the region to act as either a promoter or an enhancer (Li *et al.*, 2012). In fact, we identified 242 out of 251 RERs (96.4%) as presumptive promoters; only a small proportion of RERs corresponded to presumptive enhancers (3.6%) (Fig. 2b).

Closer inspection of the RERs located near TSSs showed that they were concentrated in the 5′ untranslated regions (5′ UTRs) and downstream of the TSSs (Fig. 2c). However, analysis of the location of peaks at the different stages showed that less than 55% of normal H3K4me3 peaks were located within 1 kb of TSSs (Fig. 2d, *P*-value < 2.2e-14, Chi-square test).

### 3.3 Genes associated with RERs are significantly enriched in GO terms associated with mRNA splicing

**Fig. 3.**
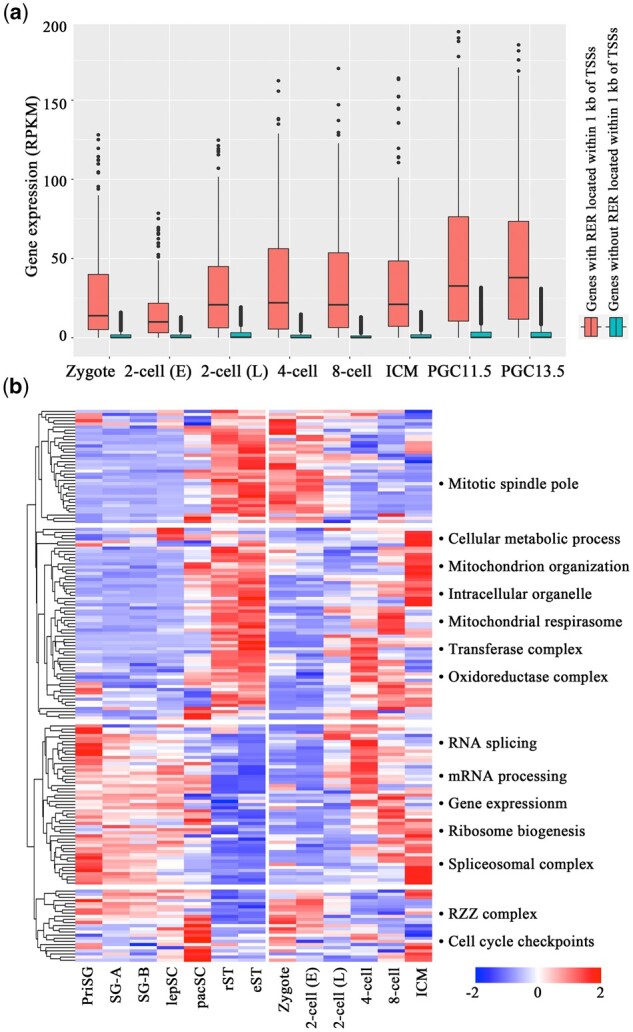
Expression patterns of the RER-associated genes. (**a**) Box plot showing the expression levels of genes with or without RERs in their promoters (±1 kb around the TSS) during embryonic development and in PGCs (*P*-value < 2.2e-16, Student's *t*-test). (**b**) Left, heat map illustrating the expression pattern of genes with RERs in their promoters (±1 kb around the TSS) during spermatogenesis and early embryonic stages. The genes whose expression is upregulated are depicted in red, and those whose expression is downregulated are shown in blue. Hierarchical cluster analysis grouped the genes into four clusters according to their expression levels at different stages. Right panel: the corresponding significantly enriched GO terms for each cluster are shown (FDR < 0.05, Fisher's exact test). priSG (primitive spermatogonia), SG-A (type-A spermatogonia), SG-B (type-B spermatogonia), lepSC (leptotene spermatocytes), pacSC (pachytene spermatocytes), rST (round spermatids), eST (elongated spermatids), 2-cell (E) (2-cell early), 2-cell (L) (2-cell late)

To explore the gene expression pattern from the testis through the early embryonic developmental stages, we used published datasets of genes that are expressed in primitive spermatogonia (PriSG), type-A spermatogonia (SG-A), type-B spermatogonia (SG-B), leptotene spermatocytes (lepSC), pachytene spermatocytes (pacSC), round spermatids (rST) and elongating spermatids (eST) (Gan *et al.*, 2013). The expression levels of approximately half of the genes were highest in spermatids, whereas the RNA expression levels of the other genes were greatest in spermatogonia and spermatocytes. In fact, all the genes could be grouped into four clusters (Fig. 3b). Notably, for the second gene cluster (spermatid-4 cell-specific), RNA expression strongly decreased from elongated spermatids to zygotes, but the expression level increased from the 4-cell stage onward. Sixty-two genes were significantly enriched in GO terms such as ‘cellular metabolic process’ (*Sucla2*, *Rpl30*, *Cstf1*, *Mms22l*, *Ercc3*, etc.) and ‘mitochondrial organization’ (*Rb1cc1*, *Timm23*, *Atg13*, *Ap3b1*, *Lig3*, etc.). For the third gene cluster (spermatogonia-spermatocyte-4 cell-specific), the expression of the genes was upregulated in spermatogonia, spermatocytes and the 4-cell stage onward, whereas the expression level decreased in spermatids, zygotes and the 2-cell stage. Interestingly, 52 genes were significantly enriched in GO terms such as ‘RNA splicing’ (*Prpf31*, *Sf3a3*, *Smndc1*, *Alyref*, *Snrnp40*, *Cwf19l1*, *Hnrnpc*, *Rbm8a*), ‘mRNA processing’ (*Pdcd11*, *Polr2d*, *Rnmt*, etc.) and ‘gene expression’ (*Abt1*, *Rps3*, *Rpl4*, *Zmpste24*, *Ddx52*, *Tpr*, *Noa1*, *Utp15*, *Imp4*, etc.).

A total of 179 genes were identified with RERs located within 1 kb of TSSs. As expected, the genes with RERs within the TSS had higher expression levels than did genes without RERs (Fig. 3a, *P*-value < 2.2e-16, Student's *t*-test).

In summary, the genes with RERs located within 1 kb of TSSs were dynamically expressed during spermatogenesis and early developmental stages. Clustering the genes according to the expression levels in different stages revealed that some of the genes were significantly enriched in GO terms such as ‘RNA splicing’, ‘mRNA processing’ and ‘gene expression’. These results suggest that the H3K4me3 marks located within the TSS of RNA splicing genes survive two rounds of the epigenetic reprogramming process. Considering that embryonic exposure to ATZ increases the expression of alternatively spliced RNA isoforms, we propose that the altered H3K4me3 marks that are located within RNA splicing genes are transferred to subsequent generations and affect the expression of alternatively spliced RNA isoforms in the third generation.

### 3.4 H3K4me3 peaks including RERs are enriched in SP1/SP2/SP3 and SRY motifs

**Fig. 4.**
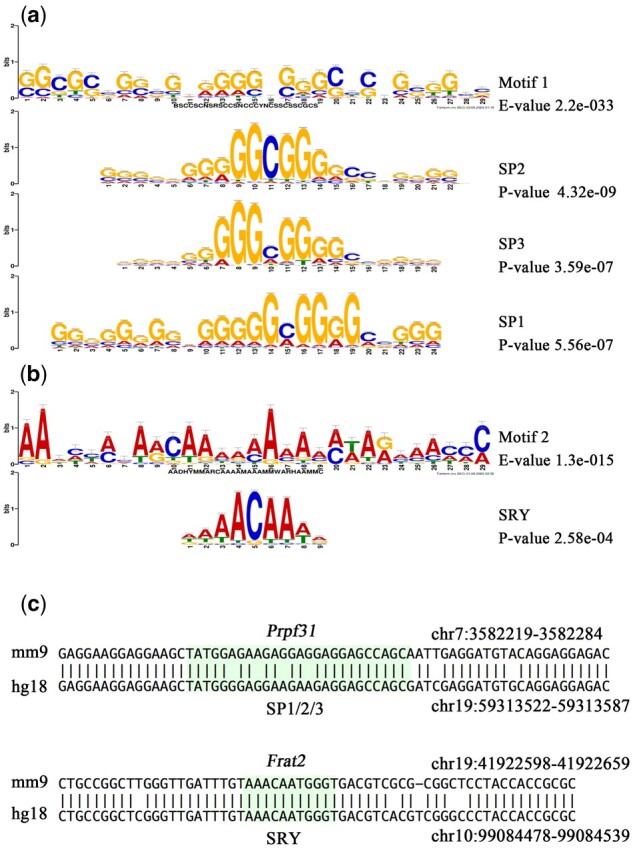
H3K4me3 peaks associated with RERs are significantly enriched in two motifs. (**a**) Four-cell stage H3K4me3 peaks associated with RERs enriched in the GC-rich motif (motif 1, the enriched E-value is shown on the right). The SP2-, SP3- and SP1-binding sites are shown below the motif (the comparison *P*-value generated by TOMTOM is shown on the right). (**b**) PGC11.5 stage H3K4me3 peaks associated with RERs enriched in the AT-rich motif (motif 2). The SRY-binding site is shown below the motif. (**c**) Conserved binding sites located within the *Prpf31* and *Frat2* genes; the conserved region containing the binding sites for SP1/SP2/SP3/SRY is highlighted in green

To explore the role of the transcriptional control of RERs, motif enrichment with MEME-ChIP was applied to the H3K4me3 peaks with RERs at 9 developmental stages (sperm, PN5 zygotes, the early two-cell stage, the late two-cell stage, the four-cell stage, eight-cell embryos, ICM, E11.5 PGCs and E13.5 PGCs). As expected, a similar significantly enriched GC-rich motif (motif 1) emerged within the sequences of H3K4me3 peaks from sperm to ICMs (MEME-ChIP E-value < 2.6e-07, Supplementary Fig. S2). As an example, the motif enriched by 4-cell stage H3K4me3 peaks is shown in Figure 4a. The investigation of this motif via TomTom (Gupta *et al.*, 2007) revealed parts of the motif that strongly resemble the binding sites for the specificity protein (SP) group (SP1, SP2 and SP3) of transcriptional factors (the most significant cases, TomTom *P*-value < 1.33e-04). The binding sites for these factors were GC-rich DNA sequences (Fig. 4a, Supplementary Fig. S2).

Remarkably, the RER-associated H3K4me3 peaks in PGC 11.5 and PGC 13.5 were significantly enriched AT-rich motifs (motif 2, MEME-ChIP E-value < 2.0e-08; Fig. 4b). A portion of these motifs strongly matched the binding site for SRY (the most significant case, *P*-value < 2.58e-04), which is a key regulator of the development of male gonads and is critical for normal male sex determination (Fig. 4b) (Kashimada and Koopman, 2010).

To further explore the involvement of these factors in gene network regulation, we identified RER-associated genes with binding sites of motifs (SP1, SP2, SP3 and SRY) that emerged within the promoters. By using FIMO, we identified conserved motif-binding sites within the RER-associated H3K4me3 peaks (Grant *et al.*, 2011). The SP1, SP2 and SP3 motifs are present in promoters of genes associated with RNA splicing (*Prpf31, Smndc1, Alyref, Snrnp40, Hnrnpc, Rbm8a*). For example, conserved blocks were present within the promoter of *Prpf31*, which contains binding sites for the SP1/SP3/SP2 factors (Fig. 4c). There are eight genes (*Jam2, Zfp830, Atg13, Frat2, Harbi1, Kti12, Rnf125, A730015C16Rik*) with conserved binding sites for the SRY motif. In addition, the promoter of the *Frat2* gene involves a binding site for SRY (Fig. 4c).

In summary, our data show that RER-related H3K4me3 peaks were enriched in SP1, SP2, SP3 and SRY transcription factor binding sites. We propose that the epigenetic changes in many genes are mediated by the action of members of the SP family and SRY transcription factors.

### 3.5 Embryonic exposure to ATZ is associated with differential RNA expression of RER-related genes in the third generation

In our previous publication, gestating mice were treated with ATZ during the embryonic period from E6.5 to E15.5. Genome-wide transcriptomic analysis of the testis, liver and brain was performed in the F3 generation via high-throughput RNA-seq. In the current study, to reveal whether gestational exposure to ATZ will affect the RNA expression of RER-related genes in the third generation, the transcripts assigned to the 179 RER-related genes were selected for further analysis. By analysing the RNA-seq data in the F3 generation, we detected 11 (*1700064H15Rik*, *Ankra2*, *Atg16l1*, *Ccnc*, *Cdc123*, *Ercc8*, *Foxred1*, *Iqck*, *Ndufs5*, *Ttc39b*, *Zfp933*), 6 (*Aldh6a1*, *Ccdc17*, *Ercc8*, *Got1*, *Rnf167*, *Timm23*) and 2 (*Cse1l*, *Ergic2*) differentially expressed genes in the testis, liver and brain [fold change (FC)>2 and FDR < 0.1].

The differentially expressed genes in the testis encode proteins that function in the regulation of transcription, such as *Ankra2*, *Ccnc*, *Cdc123* and *Ercc8*.

Notably, *Ercc8* was significantly differentially expressed in both the testis and liver. However, the expression level in the testis decreased in ATZ-lineage males, while the level of RNA expression in the liver increased. The Ercc8 gene provides instructions for making a protein called Cockayne syndrome A (CSA), which is involved in repairing damaged DNA. The dysregulation of this gene in both the testis and liver indicates that the Ercc8 gene may be vulnerable to ATZ embryonic exposure, and the effects persist to the offspring.

These data suggest that embryonic exposure to ATZ can affect the expression of RER-related genes in many tissues of F3-generation offspring.

## 4 Discussion

In the current study, based on H3K4me3 ChIP-seq data of mouse sperm, early embryonic development and PGCs, RERs were detected as regions with H3K4me3 marks retained in sperm, and the signal escaped epigenetic reprogramming of the paternal genome during early embryonic development and male gonad PGC development.

Interestingly, the expression levels of the *Prpf31* gene, which is involved in pre-mRNA splicing as a component of the spliceosome, were also slightly but significantly increased in ATZ-lineage F3 male testes (FC = 1.2, *P*-value = 0.04, *t*-test). The expression levels of the *Snrnp40* gene, which is required for pre-mRNA splicing, were significantly decreased in ATZ-lineage F3 male testes (FC = 1.2, *P*-value = 0.07, *t*-test).

It was reported in our previous research that embryonic exposure to ATZ was associated with increases in the appearance of the new isoforms that were initiated from alternative TSSs or splicing. In the current study, RER-related genes were found to encode proteins that function in mRNA splicing and transcriptional regulation. Combined with the RNA-seq data of testes in the F3 generation from our previously published transgenerational study, the data of the differentially expressed RER-related genes also revealed involvement in alternative splicing and regulation of transcription. These results suggest that genes with functions in alternative splicing and transcriptional regulation harbour H3K4me3 marks in sperm, which can escape two rounds of epigenetic reprogramming during embryonic development. Upon environmental factors that alter the H3K4me3 marks within these genes, the effects may be transmitted to subsequent generations. In the current study, after embryonic exposure to ATZ, the expression levels of RER genes that function in mRNA alternative splicing and transcriptional regulation were altered. These results may explain the increased appearance of new isoforms triggered by alternative TSSs and the expression of alternatively spliced RNA isoforms in the F3 generation.

The existence of SP family member-binding sites in many RER-related H3K4me3 peaks, especially genes that function in alternative splicing, suggests a potential role in the epigenetic regulation of genes. It was demonstrated that transcription factors of the SP family play critical roles in the early development of mice (Safe *et al.*, 2014). Remarkably, the results of our previous ATZ project showed that SP family member-binding sites are present within many promoters with altered TSSs and in many altered H3K4me3 peaks in the F3 generation. These data indicate that SP family transcription factors may be involved in mediating the establishment of H3K4me3 occupancy in RERs and may be involved in the regulation of alternatively spliced transcript expression.

Several studies by the Skinner laboratory using different chemical compounds showed that exposure during a critical reprogramming window in F0 leads to transgenerational effects in F3 generation(Anway, 2005; Manikkam *et al.*, 2012, 2013, 2014; Skinner *et al.*, 2013; Tracey *et al.*, 2013). Remarkably, these studies revealed that DNA methylation in sperm of subsequent generations was altered. Two motifs were identified that are associated with the F3 sperm differential DNA methylation regions (DMR) from different environmental exposures. Two different motifs were identified: an AT-rich motif that is present in the transgenerational vinclozolin DMR set, and a GC-rich motif present in the other germline transgenerational DMR sets investigated (jet fuel, pesticides, plastics and dioxin; Guerrero-Bosagna *et al.*, 2014). In the current study, RER-associated H3K4me3 peaks were significantly enriched in similar AT-rich and GC-rich motifs as were identified from DMR. Although in theory any part of the genome could be affected in response to experiences or exposure, these data suggest that some regions are more prone to be environment-sensitive than others. The AT-rich and GC-rich DNA sequence motifs may promote a region of sensitivity for these RERs or DMRs to be programmed transgenerationally.

## Supplementary Material

btaa920_Supplementary_Data
