# Supplementary material for: Paternal reprogramming-escape histone H3K4me3 marks located within promoters of RNA splicing genes
Source: Bioinformatics. 2020 Nov 23;37(8):1039–44. doi: 10.1093/bioinformatics/btaa920 (PMC8150124; doi:10.1093/bioinformatics/btaa920)
Supplement: btaa920_Supplementary_Data [file btaa920_supplementary_data.zip › Supplemental Legends.docx]

**Supplementary Figure 1.** Width of H3K4me3 peaks and RERs. (a) Violin plots representing the width of H3K4me3 peaks in different stages. (b) Violin plots illustrating the width of retained regions in the early stages and RERs. (left: the width of regions with H3K4me3 marks present in sperm, zygotes, the 2-cell early stage, the 2-cell late stage, the 4-cell stage, the 8-cell stage and ICMs)

**Supplementary Figure 2** The H3K4me3 peaks associated with RERs in different stages are significantly enriched in similar motifs. The enriched motif at each stage and the SP2/SRY-binding sites are shown. (The enriched E-value and the comparison p-value are shown on the right.)

**Supplementary Table 1**: Coordinates of 203 regions and the associated genes. The regions with H3K4me3 marks shown in sperm and 6 early developmental stages (zygotes, the two-cell stage (early and late), the four-cell stage, eight-cell embryos and ICMs from blastocysts).

**Supplementary Table 2**: Coordinates of 251 RERs and the associated genes. The regions with H3K4me3 marks shown in sperm, 6 early developmental stages (zygotes, the two-cell stage (early and late), the four-cell stage, eight-cell embryos and ICMs from blastocysts), PGC11.5 and PGC13.5.
