# Supplementary material for: Paternal reprogramming-escape histone H3K4me3 marks located within promoters of RNA splicing genes
Source: Bioinformatics. 2020 Nov 23;37(8):1039–44. doi: 10.1093/bioinformatics/btaa920 (PMC8150124; doi:10.1093/bioinformatics/btaa920)
Supplement: btaa920_Supplementary_Data [file btaa920_supplementary_data.zip › Supplementary Table 1.pdf]

**Supplementary Table 1**: Coordinates of 203 regions and the associated genes.			
chromosome	start	end	Gene (distance to TSS)
chr1	6204461	6204586	Rb1cc1 (-202)
chr1	16645107	16646392	NONE
chr1	34496333	34497238	Ccdc115 (-269), Imp4 (+90)
chr1	54307073	54308196	Ccdc150 (+108)
chr1	55082919	55083748	Sf3b1 (+988)
chr1	55084802	55084941	Sf3b1 (-550)
chr1	57434017	57434928	1700066M21Rik (+9)
chr1	71148761	71149476	Bard1 (+601)
chr1	78484901	78486211	Farsb (-122)
chr1	89652115	89653257	Atg16l1 (+25)
chr1	90174318	90175308	Hjurp (-605)
chr1	121422148	121423506	NONE
chr1	121809142	121809386	Tmem177 (+521)
chr1	136390775	136392647	Rabif (+486)
chr1	141351099	141351940	Aspm (+168)
chr1	152239433	152241211	BC003331 (-112), Tpr (-58)
chr1	157888066	157889826	NONE
chr1	182185933	182187105	Psen2 (-88)
chr10	29919468	29920425	Cenpw (+399)
chr10	59464918	59466421	Ascc1 (+83), Anapc16 (+190)
chr10	62891453	62892721	Ctnna3 (-759)
chr10	67011850	67012644	Ado (-544)
chr10	75700220	75701144	Prmt2 (-101)
chr10	105278273	105278982	Mettl25 (-194), Ccdc59 (+505)
chr10	116661651	116662638	Yeats4 (-582)
chr10	118675601	118676065	NONE
chr11	22890208	22891233	Cct4 (+202)
chr11	34646465	34646943	Spdl1 (+439)
chr11	40546474	40548051	Hmmr (-339), Nudcd2 (+94)
chr11	57641035	57641855	NONE

chr11	62364149	62365574	Ubb (+189)
chr11	70460065	70461930	Slc25a11 (-438), Rnf167 (+209)
chr11	72020637	72021961	Txndc17 (+250)
chr11	82577925	82579129	Cct6b (-719), Zfp830 (+680)
chr11	82593657	82595939	Lig3 (+188)
chr11	83755144	83756130	Ddx52 (+73)
chr11	117709355	117711025	Birc5 (-375)
chr11	120460151	120461213	Anapc11 (+836)
chr12	53703309	53704937	NONE
chr12	66174303	66176361	Fkbp3 (-401)
chr12	80189991	80190893	Pigh (+215)
chr12	85791126	85791807	Lin52 (-991), Aldh6a1 (+433)
chr12	85792820	85793051	Lin52 (+478)
chr12	103150595	103151907	Trip11 (+226)
chr12	104662676	104664103	Ddx24 (+600)
chr12	110131437	110133043	Wars (-833), Wdr25 (-242)
chr13	22086828	22088206	NONE
chr13	22112838	22113784	NONE
chr13	23515175	23515986	Abt1 (+154)
chr13	23854708	23855719	Hist1h3a (-959), Hist1h1a (-323)
chr13	39051964	39052030	Slc35b3 (+747)
chr13	95128415	95129644	Ap3b1 (+115)
chr13	99032527	99032933	Ankra2 (-467), Utp15 (+217)
chr13	105017924	105019463	Cenpk (-774), Ppwd1 (+230)
chr13	108947973	108949059	Ercc8 (-416), Ndufaf2 (+303)
chr14	33015978	33016108	Timm23 (-959), Parg (+908)
chr14	52722499	52723397	Hnrnpc (+731)
chr14	54872722	54874355	Dad1 (+240)
chr14	54878989	54879645	Abhd4 (+410)
chr14	73952261	73954272	Sucla2 (+795)
chr15	34372096	34372739	Rpl30 (+977)
chr15	54922265	54922761	Dscc1 (-467)

chr15	72640557	72640708	NONE
chr15	81641009	81642602	Tef (-38)
chr16	22857251	22857501	Dnajb11 (-542), Tbccd1 (+266)
chr16	84775345	84775479	NONE
chr16	87495338	87495821	Cct8 (+538)
chr16	91098334	91098804	NONE
chr17	7031454	7032079	NONE
chr17	29170407	29171658	NONE
chr18	21120551	21122007	NONE
chr18	31948395	31949476	Polr2d (+123)
chr18	32399264	32400974	Ercc3 (+165)
chr18	34165120	34165133	NONE
chr18	34910714	34911861	Cdc25c (-101)
chr18	36903441	36904779	Ik (-200), Ndufa2 (+92)
chr18	46439634	46440826	Pggt1b (+274)
chr18	63137060	63137809	Napg (-55)
chr18	68459205	68460912	Fam210a (-72), Rnmt (+50)
chr18	74938102	74939321	Acaa2 (-139)
chr19	29064397	29065219	Cdc37l1 (-176)
chr19	34996945	34997622	Kif20b (+436)
chr19	37449859	37451710	Kif11 (-108)
chr19	41922691	41923134	Frat2 (-291)
chr19	43598366	43598810	Got1 (+507)
chr19	44209229	44210246	Cwf19l1 (+628)
chr19	46378897	46379155	Nfkb2 (-201)
chr19	47123540	47125155	Pcgf6 (+987)
chr19	47163880	47165774	Pdcd11 (-429), Usmg5 (+288)
chr19	53464120	53464461	Smndc1 (+772)
chr2	3429548	3430674	Cdnf (-226)
chr2	5765543	5766516	Nudt5 (-35), Cdc123 (+180)
chr2	13995241	13996400	Stam (+94)
chr2	28915766	28917436	Ttf1 (+819)

chr2	37214082	37215136	Pdcl (+243)
chr2	52600728	52602104	Stam2 (-182)
chr2	91549826	91551581	Harbi1 (-375), Atg13 (-28)
chr2	92273240	92274303	Cry2 (+428)
chr2	139996820	139996888	Esf1 (-554), Ndufaf5 (+469)
chr2	144381030	144381674	Sec23b (-640)
chr2	162842377	162843569	Ift52 (-117)
chr2	166730727	166733378	Cse1l (+494)
chr2	172194915	172195667	Cstf1 (-943), Aurka (+715)
chr2	172265771	172267274	Rtfdc1 (+467)
chr3	19527413	19529544	1700064H15Rik (+198)
chr3	30894471	30895438	Prkci (+286)
chr3	65759978	65761021	NONE
chr3	65763056	65763274	Ccnl1 (-994)
chr3	94690738	94691163	Psmb4 (-68)
chr3	96433544	96434490	Rbm8a (+166)
chr3	105761192	105761893	Wdr77 (-744)
chr3	105763101	105763827	Atp5f1 (-447)
chr3	108374730	108376992	NONE
chr3	116297348	116298585	Sass6 (+41)
chr4	8574063	8574064	NONE
chr4	8574941	8575697	NONE
chr4	21654329	21655630	Ccnc (+107)
chr4	24423359	24424152	Mms22l (+158)
chr4	34560852	34562915	Rars2 (-322), Orc3 (+309)
chr4	35171859	35173832	3110043O21Rik (+283)
chr4	40895901	40896352	Bag1 (-851), Chmp5 (+687)
chr4	45420882	45421939	Slc25a51 (+222)
chr4	47486625	47487919	Sec61b (-258), Alg2 (-67)
chr4	49533352	49534358	Zfp189 (-234), Mrpl50 (+110)
chr4	82969035	82969949	Ttc39b (+667)
chr4	88367389	88368418	Klhl9 (+465)

chr4	108520593	108522168	A730015C16Rik (-157), Kti12 (+919)
chr4	116269689	116270989	NONE
chr4	116357864	116359346	Prdx1 (+456)
chr4	119164703	119165513	Rimkla (+125)
chr4	120769697	120770776	Zmpste24 (+609)
chr4	123394797	123395815	Ndufs5 (+139)
chr4	124391777	124392663	Sf3a3 (+200)
chr4	128670101	128671408	Ak2 (+287)
chr4	130036184	130038167	Snrnp40 (-203), Zcchc17 (+14)
chr4	140795109	140795829	NONE
chr4	147221922	147223089	Zfp933 (-31)
chr5	20930885	20931563	Ccdc146 (-729), Fam185a (+448)
chr5	30061475	30065108	NONE
chr5	34278432	34279479	Nelfa (+106)
chr5	46060252	46061654	Ncapg (-208)
chr5	74931577	74932739	Fip1l1 (+651)
chr5	77738013	77739911	Polr2b (-210), Noa1 (+147)
chr5	92390960	92392637	Rchy1 (+295)
chr5	115790652	115792169	Gatc (-224), Triap1 (+177)
chr5	118695319	118695721	Rnft2 (-650), 2410131K14Rik (+284)
chr5	124198967	124199195	Kntc1 (-654), Rsrc2 (+340)
chr5	124200390	124200691	Kntc1 (+806)
chr5	124778204	124778873	Mphosph9 (-558), 2810006K23Rik (+432)
chr5	130362593	130363157	Chchd2 (+465)
chr5	147644088	147644327	Rpl21 (-258)
chr5	151324848	151325842	Brca2 (+140)
chr6	24477194	24477846	Asb15 (-624), Ndufa5 (+493)
chr6	29297443	29298657	Calu (-56)
chr6	39368334	39369177	NONE
chr6	39370204	39370866	Mkrn1 (-74)
chr6	47870283	47871485	Zfp212 (+409)

chr6	81991350	81992599	Eva1a (+358)
chr6	88791243	88791977	Abtb1 (+319)
chr6	120480864	120481845	Hdhd5 (-18)
chr6	128787991	128789696	NONE
chr6	148159616	148159893	NONE
chr6	148160972	148161380	Ergic2 (-280)
chr7	3581124	3582389	Tfpt (-226), Prpf31 (+170)
chr7	4101564	4102458	Leng9 (-537)
chr7	6335495	6336586	Zfp28 (-39)
chr7	16507033	16508548	Selenow (-40)
chr7	28763583	28765027	NONE
chr7	35173838	35173880	Lsm14a (+700)
chr7	36019076	36019460	NONE
chr7	50567977	50568896	NONE
chr7	52972901	52974480	Sphk2 (-419)
chr7	87679312	87680365	Blm (+45)
chr7	88045240	88046740	Wdr73 (+165)
chr7	88907372	88909316	Fam103a1 (+533)
chr7	97129435	97130462	Eed (-456)
chr7	100022092	100023504	Prcp (-965), Ddias (-55)
chr7	106631178	106632382	Rps3 (+468)
chr7	125258929	125259614	Rps15a (+428)
chr7	125998348	126000190	Knop1 (-153), Iqck (-20)
chr7	149087736	149089142	Tollip (-47)
chr8	11479483	11479889	NONE
chr8	12671181	12672917	Tubgcp3 (+199)
chr8	13784802	13785489	Upf3a (-469)
chr8	23763276	23764468	Polb (+37)
chr8	47249638	47250119	Cfap97 (+790)
chr8	63471041	63472988	Nek1 (+23)
chr8	107164583	107166141	Fam96b (+503)
chr8	114463862	114465675	Gabarapl2 (+166)

chr8	120324702	120326776	Mphosph6 (+90)
chr8	125898318	125898466	Tcf25 (+668)
chr8	128947265	128947733	NONE
chr9	7836255	7837145	NONE
chr9	13630237	13630890	Cep57 (+987)
chr9	35017791	35019649	Foxred1 (-155), Srpr (-20)
chr9	36574474	36576082	Stt3a (-14)
chr9	59504705	59504959	Pkm (+449)
chr9	64019354	64019526	NONE
chr9	64020490	64022140	Zwilch (-404), Rpl4 (+121)
chr9	64101660	64101672	Map2k1 (-228)
chr9	64808719	64809414	Ints14 (+428)
chr9	103103823	103104848	Srprb (+131)
chr9	111020185	111020398	Lrrfip2 (-323)
