# Supplementary material for: Paternal reprogramming-escape histone H3K4me3 marks located within promoters of RNA splicing genes
Source: Bioinformatics. 2020 Nov 23;37(8):1039–44. doi: 10.1093/bioinformatics/btaa920 (PMC8150124; doi:10.1093/bioinformatics/btaa920)
Supplement: btaa920_Supplementary_Data [file btaa920_supplementary_data.zip › Supplementary Table 2.pdf]

**Supplementary Table 2**: Coordinates of 251 RERs and the associated genes.			
chromosome	start	end	Gene (distance to TSS)
chr1	6204489	6204586	Rb1cc1 (-188)
chr1	34496788	34497071	Ccdc115 (-413), Imp4 (+234)
chr1	54307994	54308196	Ccdc150 (+568)
chr1	57434886	57434928	1700066M21Rik (+443)
chr1	71148787	71149028	Bard1 (+812)
chr1	78484901	78485002	Farsb (+482)
chr1	78485049	78485252	Farsb (+283)
chr1	89652115	89652347	Atg16l1 (-430)
chr1	136391296	136391836	Rabif (+341)
chr1	141351099	141351122	Aspm (-241)
chr1	141351792	141351940	Aspm (+514)
chr1	152239635	152239905	Tpr (-610), BC003331 (+440)
chr1	157888066	157888234	NONE
chr10	29919468	29919753	Cenpw (+735)
chr10	29920234	29920425	Cenpw (+16)
chr10	59465055	59465359	Ascc1 (-380), Anapc16 (+653)
chr10	59465873	59466205	Anapc16 (-179), Ascc1 (+452)
chr10	59466244	59466305	Anapc16 (-415), Ascc1 (+688)
chr10	62892626	62892721	Ctnna3 (-172)
chr10	75700226	75700548	Prmt2 (+194)
chr10	75700873	75701074	Prmt2 (-393)
chr10	116661651	116661939	Yeats4 (-232)
chr10	118675745	118675748	NONE
chr10	118675854	118676065	NONE
chr11	22890208	22890318	Cct4 (-256)
chr11	34646652	34646943	Spdl1 (+345)
chr11	40546527	40546895	Nudcd2 (-458), Hmmr (+213)
chr11	40547361	40547978	Hmmr (-746), Nudcd2 (+501)
chr11	57641060	57641323	NONE
chr11	62364149	62364597	Ubb (-300)

chr11	62365056	62365379	Ubb (+545)
chr11	62365527	62365574	Ubb (+878)
chr11	70460065	70460277	Rnf167 (-618), Slc25a11 (+389)
chr11	70460755	70460949	Slc25a11 (-292), Rnf167 (+63)
chr11	70461081	70461319	Slc25a11 (-640), Rnf167 (+411)
chr11	70461356	70461930	Rnf167 (+854)
chr11	72020812	72021053	Txndc17 (-116)
chr11	72021196	72021501	Txndc17 (+300)
chr11	82578247	82578521	Cct6b (-576), Zfp830 (+537)
chr11	82578755	82579005	NONE
chr11	82593657	82593984	Lig3 (-789)
chr11	82595122	82595939	Lig3 (+921)
chr11	83755658	83755909	Ddx52 (+220)
chr11	117710609	117710910	Birc5 (+195)
chr11	120460216	120460997	Alyref (-928), Anapc11 (+761)
chr12	53704220	53704598	NONE
chr12	66174303	66174550	Fkbp3 (+504)
chr12	66175227	66175547	Fkbp3 (-456)
chr12	80190072	80190428	Pigh (+407)
chr12	85791514	85791807	Lin52 (-797), Aldh6a1 (+239)
chr12	85792820	85793051	Lin52 (+478)
chr12	103150657	103151071	Trip11 (+613)
chr12	104663566	104663720	Ddx24 (+347)
chr12	104663761	104663997	Ddx24 (+111)
chr12	110131907	110132291	Wars (-692), Wdr25 (-383)
chr12	110132510	110132771	Wdr25 (+159)
chr13	22087245	22087703	NONE
chr13	23515175	23515433	Abt1 (+431)
chr13	95128415	95128422	Ap3b1 (-496)
chr13	95128529	95128785	Ap3b1 (-258)
chr13	95129563	95129644	Ap3b1 (+689)
chr13	99032527	99032915	Ankra2 (-476), Utp15 (+226)

chr13	108948210	108948825	Ercc8 (-414), Ndufaf2 (+301)
chr13	108949025	108949059	Ndufaf2 (-223), Ercc8 (+110)
chr14	33015978	33016108	Timm23 (-959), Parg (+908)
chr14	52722610	52722811	Hnrnpc (+968)
chr14	52722986	52723027	Hnrnpc (+672)
chr14	52723108	52723397	Hnrnpc (+426)
chr14	54873370	54873572	Dad1 (+308)
chr14	73953309	73953508	Sucla2 (+937)
chr15	34372096	34372739	Rpl30 (+977)
chr15	81641009	81641279	Tef (-700)
chr15	81642258	81642473	Tef (+522)
chr16	84775345	84775479	NONE
chr16	87495448	87495821	Cct8 (+483)
chr17	7031903	7032079	NONE
chr17	29170407	29170892	NONE
chr17	29171567	29171658	NONE
chr18	21120551	21120714	Rnf125 (+495)
chr18	21121138	21121363	NONE
chr18	31948395	31948658	Polr2d (-286)
chr18	31949099	31949476	Polr2d (+475)
chr18	32399536	32399735	Ercc3 (-318)
chr18	32399965	32400848	Ercc3 (+453)
chr18	34910714	34910834	Cdc25c (+413)
chr18	36903656	36904214	Ik (-375), Ndufa2 (+267)
chr18	36904291	36904407	Ndufa2 (-147), Ik (+39)
chr18	36904584	36904590	Ndufa2 (-385), Ik (+277)
chr18	36904640	36904779	Ndufa2 (-508), Ik (+400)
chr18	46440410	46440593	Pggt1b (+2)
chr18	63137652	63137809	Napg (+241)
chr18	68459205	68459417	Rnmt (-698), Fam210a (+676)
chr18	68459538	68459833	Rnmt (-323), Fam210a (+301)
chr18	68460091	68460630	Fam210a (-374), Rnmt (+352)

chr18	68460813	68460912	Fam210a (-876), Rnmt (+854)
chr18	74938102	74938253	Acaa2 (-673)
chr19	29064525	29064800	Cdc37l1 (-321)
chr19	29065196	29065219	Cdc37l1 (+224)
chr19	34996945	34997285	Kif20b (+267)
chr19	37450228	37450640	Kif11 (-459)
chr19	37451088	37451543	Kif11 (+423)
chr19	41922691	41922967	Frat2 (-207)
chr19	43598401	43598770	Got1 (+509)
chr19	44209693	44209868	Cwf19l1 (+585)
chr19	44209990	44210246	Cwf19l1 (+248)
chr19	47124498	47124821	Pcgf6 (+675)
chr19	47164114	47165070	Pdcd11 (-664), Usmg5 (+523)
chr19	47165321	47165699	Usmg5 (-395), Pdcd11 (+254)
chr19	53464308	53464461	Smndc1 (+678)
chr2	3429548	3430086	Cdnf (-520)
chr2	5765543	5766124	Nudt5 (-231), Cdc123 (+376)
chr2	13995241	13995428	Stam (-392)
chr2	13995734	13996051	Stam (+166)
chr2	28915829	28916449	Ttf1 (+357)
chr2	52600728	52601062	Stam2 (+339)
chr2	52601539	52602084	Stam2 (-578)
chr2	91549841	91550416	Harbi1 (-950), Atg13 (+547)
chr2	91550983	91551581	Atg13 (-606), Harbi1 (+203)
chr2	144381032	144381296	Sec23b (-828)
chr2	162842614	162842809	Ift52 (-378)
chr2	162843288	162843522	Ift52 (+315)
chr2	166731624	166731827	Cse1l (+167)
chr2	166732126	166732327	Cse1l (+668)
chr2	172194915	172195107	Aurka (+995)
chr2	172195305	172195667	Cstf1 (-748), Aurka (+520)
chr2	172266137	172266841	Rtfdc1 (+433)

chr3	19528356	19528612	1700064H15Rik (+193)
chr3	19529040	19529241	1700064H15Rik (-464)
chr3	30895022	30895438	Prkci (+561)
chr3	96433923	96434242	Rbm8a (+232)
chr3	105761196	105761893	Wdr77 (-742)
chr3	105763101	105763331	Atp5f1 (-199), Wdr77 (+929)
chr3	105763550	105763827	Atp5f1 (-672)
chr3	108374802	108374990	Taf13 (+279)
chr3	108375164	108375511	Taf13 (+721)
chr3	116298108	116298576	Sass6 (+416)
chr4	8575041	8575365	NONE
chr4	21655421	21655630	Ccnc (+653)
chr4	24423854	24424152	Mms22l (+405)
chr4	34560932	34561043	NONE
chr4	34561842	34562103	Rars2 (-233), Orc3 (+220)
chr4	35173665	35173832	3110043O21Rik (-620)
chr4	40895901	40895934	Bag1 (-642), Chmp5 (+478)
chr4	45420882	45421154	Slc25a51 (+615)
chr4	47487712	47487919	Alg2 (-611), Sec61b (+286)
chr4	49533531	49533848	Zfp189 (-399), Mrpl50 (+275)
chr4	49534115	49534358	Mrpl50 (-272), Zfp189 (+148)
chr4	82969678	82969949	Ttc39b (+345)
chr4	108520593	108520714	Kti12 (+192), A730015C16Rik (+570)
chr4	108521189	108521411	A730015C16Rik (-76), Kti12 (+838)
chr4	116269973	116270373	Ccdc17 (+838)
chr4	116357864	116358112	Prdx1 (-161)
chr4	116358408	116359007	Prdx1 (+559)
chr4	120770191	120770737	Zmpste24 (+382)
chr4	123394914	123395511	Ndufs5 (+232)
chr4	123395629	123395815	Ndufs5 (-277)

chr4	124392131	124392482	Sf3a3 (+287)
chr4	128670526	128670933	Ak2 (+262)
chr4	128671206	128671408	Ak2 (+839)
chr4	130036856	130037171	Snrnp40 (-365), Zcchc17 (+176)
chr4	130037588	130037911	Zcchc17 (-560), Snrnp40 (+371)
chr4	140795109	140795263	NONE
chr4	140795780	140795829	NONE
chr4	147221922	147222421	Zfp933 (+303)
chr4	147222747	147222970	Zfp933 (-384)
chr5	20930885	20931027	Ccdc146 (-461), Fam185a (+180)
chr5	20931072	20931339	Ccdc146 (-711), Fam185a (+430)
chr5	20931450	20931563	Fam185a (+731)
chr5	30062128	30062319	Dnajb6 (-4)
chr5	30062720	30063311	Dnajb6 (+788)
chr5	30063364	30063743	NONE
chr5	34279039	34279479	Nelfa (-197)
chr5	46060449	46060958	Ncapg (-457)
chr5	74931657	74931972	Fip1l1 (+308)
chr5	77738013	77738267	Noa1 (+969)
chr5	92391368	92391603	Rchy1 (+608)
chr5	115790689	115790959	Triap1 (-410), Gatc (+363)
chr5	115791189	115791472	Gatc (-144), Triap1 (+97)
chr5	115792046	115792169	Gatc (-921), Triap1 (+874)
chr5	118695319	118695509	Rnft2 (-544), 2410131K14Rik (+178)
chr5	124198967	124199195	Kntc1 (-654), Rsrc2 (+340)
chr5	130362593	130362682	Chchd2 (+702)
chr5	151324848	151324982	Brca2 (-290)
chr6	24477194	24477575	Asb15 (-759), Ndufa5 (+628)
chr6	29298384	29298657	Calu (+415)
chr6	39368764	39368972	NONE
chr6	39369020	39369177	NONE

chr6	39370204	39370403	Mkrn1 (+157)
chr6	81991707	81991909	Eva1a (+191)
chr6	81992372	81992599	Eva1a (+869)
chr6	88791508	88791770	Abtb1 (+290)
chr6	120481112	120481385	Hdhd5 (+88)
chr6	128788486	128788770	NONE
chr6	148159616	148159893	NONE
chr6	148160972	148161380	Ergic2 (-280)
chr7	3581124	3581230	Prpf31 (-410), Tfpt (+354)
chr7	3581778	3582168	Tfpt (-442), Prpf31 (+386)
chr7	4101564	4101788	Leng9 (-202)
chr7	4101860	4101893	Leng9 (-403)
chr7	6335495	6335711	Zfp28 (-477)
chr7	16507033	16507159	Selenow (+655)
chr7	16507450	16507694	Selenow (+179)
chr7	28764409	28764654	NONE
chr7	36019141	36019340	NONE
chr7	50567977	50568574	NONE
chr7	52972929	52973419	Sphk2 (+98)
chr7	52974438	52974480	NONE
chr7	87679392	87679670	Blm (+353)
chr7	87679756	87679979	Blm (+16)
chr7	88045830	88046041	Wdr73 (+219)
chr7	88907830	88908359	Fam103a1 (+284)
chr7	88908418	88908461	Fam103a1 (+629)
chr7	88908514	88908766	Fam103a1 (+829)
chr7	100022146	100022659	Ddias (+340)
chr7	100022911	100023221	Prcp (-697), Ddias (-323)
chr7	106631178	106631331	Rps3 (+993)
chr7	106631408	106631708	Rps3 (+690)
chr7	106631824	106632092	Rps3 (+290)
chr7	125258929	125259614	Rps15a (+428)

chr7	125998348	125998682	Iqck (-774), Knop1 (+601)
chr7	125998721	125999066	Iqck (-395), Knop1 (+222)
chr7	125999639	125999838	Knop1 (-623), Iqck (+450)
chr7	149088468	149088720	Tollip (-202)
chr8	12671462	12671794	Tubgcp3 (+620)
chr8	13785274	13785473	Upf3a (-241)
chr8	23763276	23763440	Polb (+551)
chr8	23763997	23764362	Polb (-271)
chr8	47249934	47250119	Cfap97 (+938)
chr8	107164835	107164853	NONE
chr8	107164903	107165417	Fam96b (+705)
chr8	107165695	107166016	Fam96b (+9)
chr8	114464091	114464363	Gabarapl2 (-376)
chr8	114464930	114465160	Gabarapl2 (+442)
chr8	120324702	120324884	NONE
chr8	120325290	120325737	Mphosph6 (+315)
chr8	125898318	125898457	Tcf25 (+664)
chr9	7836372	7836642	NONE
chr9	13630758	13630890	Cep57 (+727)
chr9	35017810	35018009	Srpr (-830), Foxred1 (+655)
chr9	35018782	35019158	Foxred1 (-405), Srpr (+230)
chr9	35019267	35019302	Foxred1 (-720), Srpr (+545)
chr9	35019425	35019649	Foxred1 (-972), Srpr (+797)
chr9	36574474	36575152	Stt3a (+451)
chr9	36575913	36576082	Stt3a (-734)
chr9	59504705	59504782	Pkm (+361)
chr9	64020490	64020705	Rpl4 (-596), Zwilch (+313)
chr9	64021361	64021566	Zwilch (-553), Rpl4 (+270)
chr9	64021784	64022023	Zwilch (-993), Rpl4 (+710)
chr9	64808743	64809007	Ints14 (+236)
chr9	103103996	103104176	Srprb (+381)
chr9	111020185	111020397	Lrrfip2 (-324)
