# Supplementary figures and images for: Paternal reprogramming-escape histone H3K4me3 marks located within promoters of RNA splicing genes
Source: Bioinformatics. 2020 Nov 23;37(8):1039–44. doi: 10.1093/bioinformatics/btaa920 (PMC8150124; doi:10.1093/bioinformatics/btaa920)

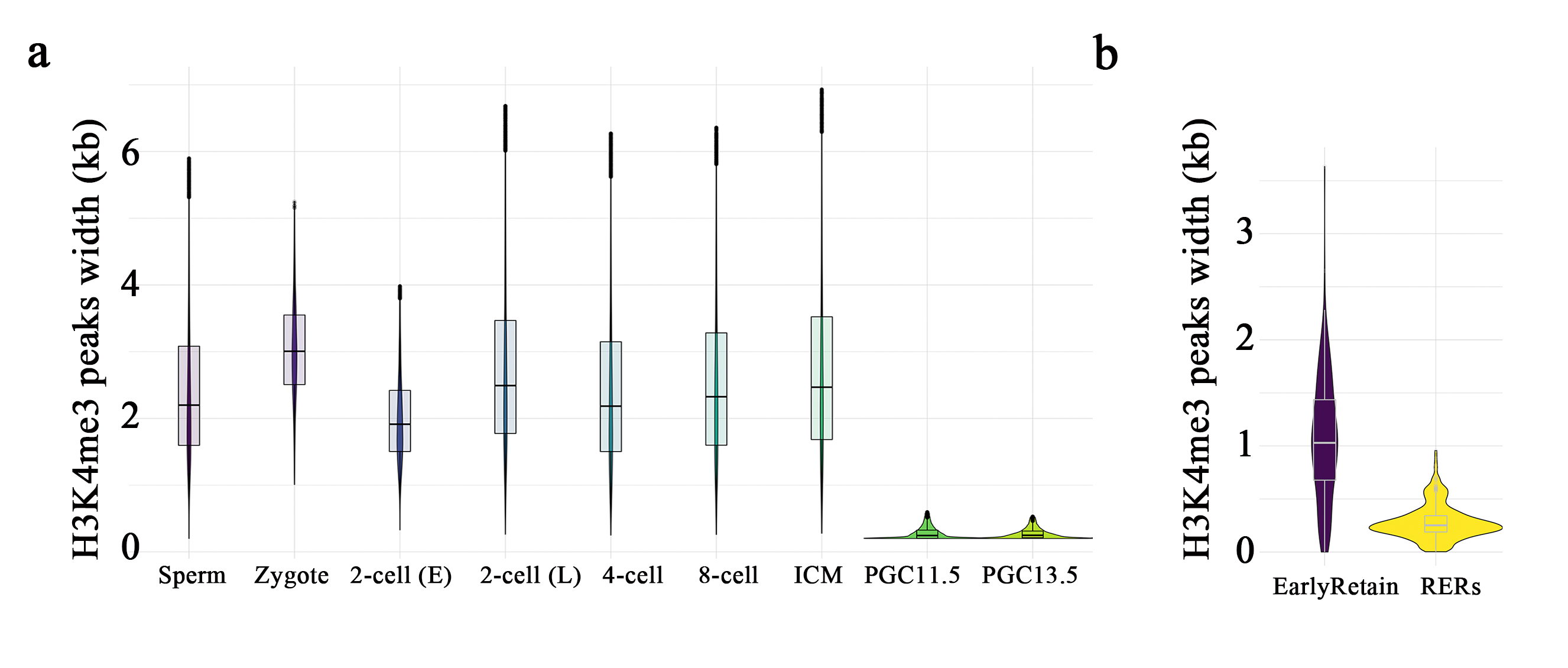

Supplement: btaa920_Supplementary_Data [file btaa920_supplementary_data.zip › suppfig1.tif]

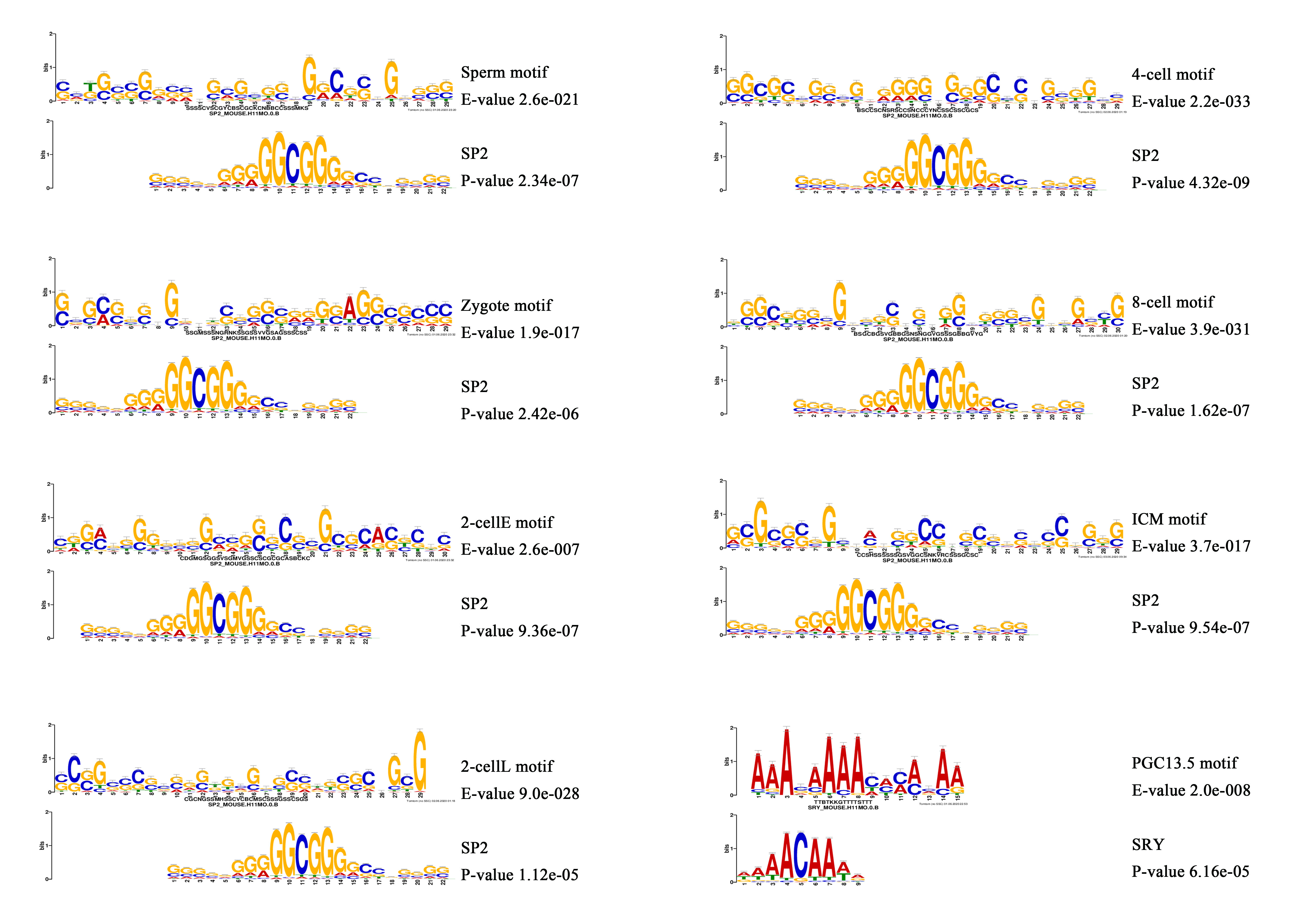

Supplement: btaa920_Supplementary_Data [file btaa920_supplementary_data.zip › suppFigure2.tif]
